# VO_2_-based switchable radiator for spacecraft thermal control

**DOI:** 10.1038/s41598-019-47572-z

**Published:** 2019-08-05

**Authors:** Heungsoo Kim, Kwok Cheung, Raymond C. Y. Auyeung, Donald E. Wilson, Kristin M. Charipar, Alberto Piqué, Nicholas A. Charipar

**Affiliations:** Naval Research Laboratory, Washington, DC 20375 USA

**Keywords:** Optical materials, Microresonators, Nonlinear optics

## Abstract

Direct calorimetric measurements of a solid state passive switchable radiator for spacecraft thermal control have been performed in a simulated space environment. Dynamic emissivity control is provided by the thermochromic phase change in a multilayer VO_2_ thin film based resonant absorber. The measured radiated power difference between 300 K and 373 K was 480 W/m^2^ corresponding to a 7× difference in radiative cooling power. We present theoretical and experimental radiator values for both normal and hemispherical as well the optical properties of VO_2_ as determined via infrared spectroscopic ellipsometry.

## Introduction

Thermal control is essential for the proper operation and longevity of space borne assets. Spacecraft thermal management systems often cope with the transient nature of thermal environments in space, such as orbital eclipses, seasonal changes in solar intensity and sun angle variation, as well as dynamic internal thermal loads (e.g. electronics, sensors, propulsion, etc.). Radiative heat transfer through a spacecraft’s thermal radiator into deep space is the sole mode of heat rejection. The thermal radiator alone is not capable of regulating the temperature but often requires supplemental heaters, heat pipes and control systems to maintain the spacecraft temperature within a desirable range^1,2^. An alternative approach is to dynamically control the emittance of the radiator via macro and micro mechanical louvers^3–6^; however, these louvers are sizable for small satellites and are susceptible to mechanical wear. Therefore, the development of cost effective, lightweight, high reliability thermal control systems (TCS) are critical for successful spacecraft missions.

Ideally, a more direct approach would modulate the emissivity of the radiator itself such that when the spacecraft temperature is lower than ideal, the emissivity is decreased to reduce radiative heat loss and when the spacecraft temperature is higher than ideal, the emissivity is increased to radiate the unnecessary heat, thus maintaining a stable spacecraft temperature. To address this matter, electrochromic^7^ and thermochromic^8,9^ variable emittance coatings have been proposed to provide efficient thermal control for spacecraft. For this work, we focus on phase change thermochromics because they do not require the use of electrical stimulus. Thin-film-based, passive TCS have been reported based on phase change materials such as chalcogenides, perovskite oxides, and vanadium dioxide (VO_2_), which can provide temperature-switchable emissivity. Chalcogenide phase change materials such as Ge_2_Sb_2_Te_5_ (GST) have been proposed to provide highly switchable emissivity^10–12^. The non-volatile switching nature between the amorphous GST and crystalline GST is ideal from a thermal control standpoint but the high temperature (913 K) necessary for crystallization requires additional power and control systems. Phase-changing materials such as perovskites and VO_2_ are more favorable because their structural phase transitions occur at lower temperatures offering the possibility of a totally passive self-regulating thermal control system. Switchable emittance was demonstrated using the perovskite oxide La_0.7_Ca_0.2_Sr_0.1_MnO_3_^13–15^; however, the width of the temperature range during the phase transition was too large (~200 K) to be an effective thermal control device.

VO_2_ is a thermochromic material well suited for TCS applications because of its dramatic change in optical properties near its phase change temperature, T_C_ ~340 K^16–18^. When T < T_C_, VO_2_ exhibits a monoclinic phase structure with an insulating state that is transparent to infrared (IR) radiation. When T > T_C_, the VO_2_ phase switches to a tetragonal phase with metallic properties and becomes IR reflecting^19,20^. Upon the transition to the metallic state (T > T_C_), its reflectance in the mid-infrared (IR) wavelength region increases and, accordingly, its emittance (ε) decreases (Δε <0) making VO_2_ suitable for smart window applications^21–24^. However, the opposite behavior (increase in emittance with increasing temperature) is required in order for VO_2_ to be used for spacecraft thermal control applications.

There have been many efforts aimed at achieving a positive, large emissivity change in VO_2_-based TCS. However, while the majority of these efforts have been theoretical studies, only a handful of experimental demonstrations have been reported. For example, it was reported that a VO_2_ film grown on an aluminum substrate showed a positive emittance change of Δε = 0.22, which is too low to be an efficient TCS^6^. Recently, Wu *et al*. provided a theoretical analysis of thermal homeostasis based on VO_2_ coated on a textured Si substrate for a switchable thermal emitter with a 10x emission change between the insulating and metallic states^25^. Hendaoui *et al*. reported a sandwich-like multilayer structure that consists of an 850 nm-thick SiO_2_ film sandwiched between a front VO_2_ thin film (30 nm) and a back infrared reflecting Au layer (350 nm) deposited on a quartz substrate. This multilayer structure provides a near normal emissivity change of Δε = 0.49 between 300 K and 373 K^19,26^. However, this multilayer structure can be degraded due to the environment in outer space. For example, the VO_2_ front layer will slowly oxidize into highly-valent V_2_O_5_ when exposed to atomic oxygen flux conditions, which greatly deteriorates its thermochromic properties^9^.

In this work, we provide direct measurements of emitted power from a VO_2_-based Fabry-Perot thin film radiator in a simulated space environment. This is in stark contrast to the bulk of previous published results which infer radiator performance from near normal optical measurements, rather than performing a direct power measurement^27^. In addition, most previously published work report near normal reflectance values which result in normal emittance values only^28^. These results are inadequate because they do not take into account the angular dependence of emissivity. Because of this, hemispherical emittance is required to fully and accurately describe the system. Rather than calculating the hemispherical emittance from reflectance measurements, which is difficult due to the number of angles, polarizations, and grazing incidences, we use a calorimetry method to directly measure the radiated power from which the emissivity can be extracted.

## Multilayer Design

The multilayer radiator design consists of a BaF_2_ dielectric spacer placed between a VO_2_/Si front layer and a gold reflecting back layer. There are several advantages to this design. By growing the VO_2_ first, we can ensure deposition of high quality VO_2_ films by optimizing the high temperature growth conditions while minimizing any issues related to thermal expansion at the interfaces between the BaF_2_ and Au layers. In addition, the Si substrate, a good IR transparent material, can protect the multilayer coatings from the external environment including exposure to atomic oxygen flux leading to a longer device lifetime. Furthermore, the Au metal layer serves as the bottom contact with the spacecraft, resulting in better thermal contact to the surface of the spacecraft, which is essential for a good thermal radiator. Finally, BaF_2_ was chosen as a dielectric spacer because of its broadband transparency in the mid-infrared region up to 15 µm. Influence of the thickness of VO_2_ and BaF_2_ layers on the emittance variation and the temperature dependence of the emitted power were studied.

**Figure 1:**
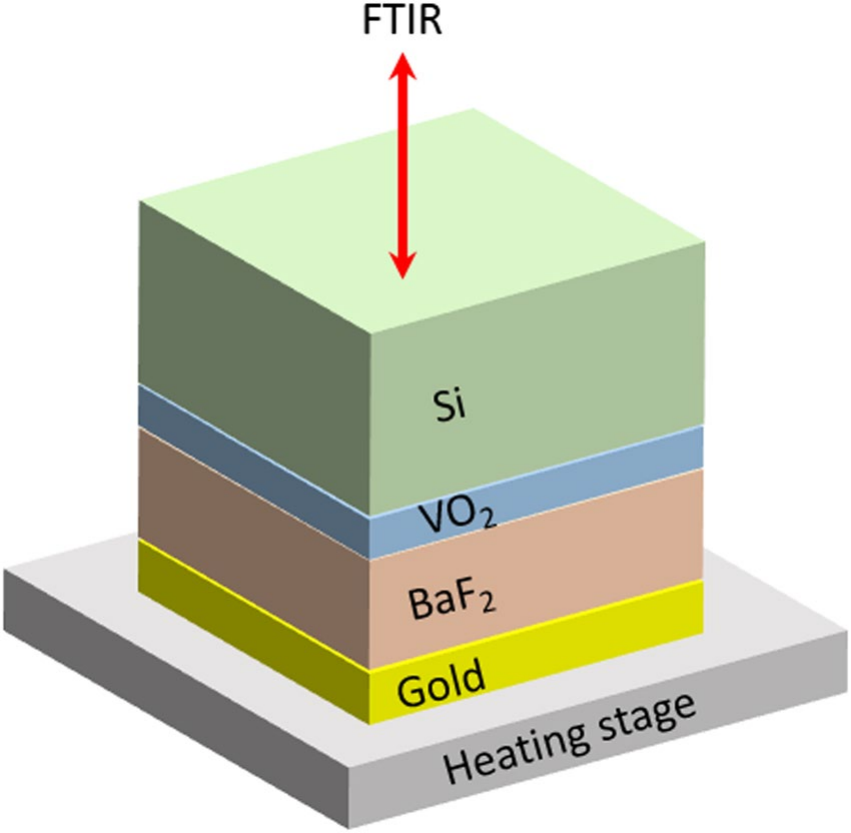
Schematic illustration of the multilayer structure mounted upside down on the temperature controlled stage inside an FTIR with insulating or metallic VO_2_. The BaF_2_ spacer (1.5 µm) and gold layer (200 nm) were deposited on the VO_2_ layer (t = 40 nm, 60 nm, 80 nm and 100 nm) on a silicon substrate.

Figure 1 shows the multilayer structure (Si/VO_2_/BaF_2_/Au) with the insulating (metallic) VO_2_ layer at 300 K (373 K). When the temperature is below 340 K, the multilayer structure is highly reflective in the mid infrared due to the bottom Au layer, since the insulating VO_2_ and the BaF_2_ layers are highly transparent in the mid infrared region. However, when the temperature is increased above 340 K, the VO_2_ becomes metallic and serves as a top mirror for a Fabry-Perot resonant structure, generating an enhanced absorption (i.e., enhanced emissivity) in the mid-infrared region, thus increasing radiative cooling. In general, the resonance wavelength in this Fabry-Perot structure can be tuned by adjusting the spacer thickness. In addition, the amplitude and the width of the resonance can also be tuned by the top mirror thickness. Thus, the thickness of the BaF_2_ spacer and the VO_2_ top mirror were chosen to achieve a maximum interference effect in the mid-infrared region (8–12 µm) corresponding to the range of the maximum emission of blackbody radiation around 300–373 K, providing enhanced emissivity in the mid-infrared region.

## Results and Discussion

### Growth and characterization of VO_2_ films

**Figure 2:**
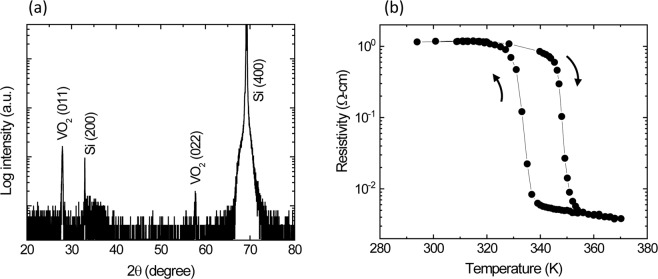
(**a**) XRD pattern and (**b**) electrical resistivity vs. temperature for 40 nm thick VO_2_ thin film grown on a Si substrate.

It is well known that the synthesis of high quality stoichiometric VO_2_ films is challenging due to the multiple stoichiometries of vanadium oxides with multivalent vanadium cations (e.g., V^2+^, V^3+^, V^4+^ and V^5+^). Available reports on the electrical and optical switching properties (i.e., the resistivity change and infrared transmittance change) for VO_2_ films are not consistent. This inconsistency indicates that the film properties are greatly influenced by the film growth conditions. Figure 2(a) shows the θ/2θ XRD scan of a 40 nm thick VO_2_ film grown on a Si (100) substrate. The peaks at ~32.9° and 69.1° are attributed to the (200) and (400) planes of the Si substrate while the peaks at ~27.9° and ~57.5° correspond to the (011) and (022) planes of monoclinic VO_2_ (M) [JCPDF Card 76-0456] respectively. Thus, it is clear that the VO_2_ film is a highly (011) oriented crystalline and single phase VO_2_ structure. These stoichiometric VO_2_ films exhibit a significant drop in resistivity and IR transmittance as the temperature increases above the T_C_ ~ 340 K. (Fig. 2b). It should be noted that a transition temperature of ~340 K is high for electronics cooling in small satellites; however, 340 K is still suitable for other subsystems such as solar arrays and reflectors. In cases where a lower transition temperature is required, it would be advantageous to dope or strain the VO_2_ thin film to reduce the transition temperature^19,29^.

### Simulated reflectance of multilayer structures

**Figure 3:**
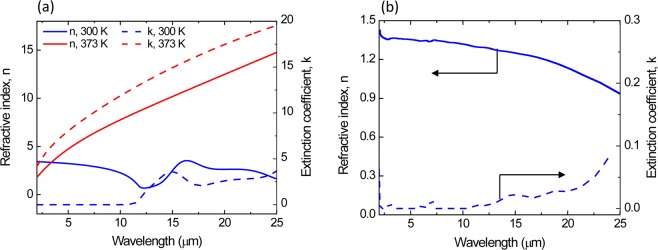
(**a**) Refractive index, *n* and extinction coefficient, *k* of a 40 nm thick VO_2_ film as a function of wavelength measured at 300 K and 373 K. (**b**) Refractive index, *n* and extinction coefficient, *k* of 1600 nm thick BaF_2_ film as a function of wavelength (2–25 µm).

**Figure 4:**
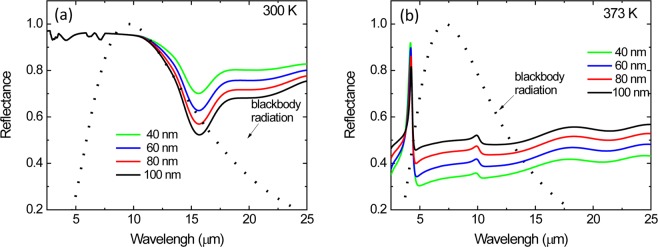
*Simulated* reflectance spectra of VO_2_-based multilayer structures with various VO_2_ thicknesses (40, 60, 80, and 100 nm) at (**a**) 300 K and (**b**) 373 K.

The optical constants (*n* and *k*) of an 80 nm VO_2_ and 1600 nm BaF_2_ were obtained from spectroscopic ellipsometry measurements by fitting the ellipsometric parameters (Ψ and Δ). Figure 3(a,b) show the optical constants of VO_2_ and BaF_2_, respectively, as a function of wavelength (2–25 µm) at 300 K and 373 K. The range of *n* and *k* values achieved in this work are similar to those previously reported by other groups for VO_2_ films grown on quartz (SiO_2_)^6^. Prior to device fabrication, the simulated reflectance spectra of the multilayer structure (Si/VO_2_/BaF_2_/Au) were generated as a function of VO_2_ layer thickness using the optical constants (*n*, *k*) data derived from ellipsometry measurements for both VO_2_ and BaF_2_ films. The BaF_2_ thickness used in these simulations is 1500 nm, which is determined to satisfy the Fabry-Perot condition of *t* = *(mλ*_*RES*_*) / (4n)* at *λ*_*RES*_ = 10 µm, where *m* is an odd integer and *n* is the refractive index of BaF_2_. Figure 4 shows the variation of simulated reflectance spectra in the infrared wavelength region (2–25 µm) for these multilayer structures (Si/VO_2_/BaF_2_/Au) at two different temperatures (300 and 373 K). The VO_2_ layer thickness was adjusted because it is common for the optical constants to vary for thin films due to density, roughness, and phase purity. The structure consists of a 40 to 100 nm-thick VO_2_ layer, a 1500 nm-thick BaF_2_ layer and a 200 nm-thick Au reflecting layer on a Si substrate. As seen in Fig. 4(a), the reflectance spectra of multilayer structures at 300 K show slight variation in the 8–10 µm range as the VO_2_ film thickness changes from 40 to 100 nm. However, at 373 K, the variation of the reflectance spectra is clearly seen when the VO_2_ becomes metallic and is semi reflective in the infrared, which forms an interference filter with the BaF_2_ and underlying Au layer. The normal infrared emittance variation (Δε) of these multilayer structures between these two temperatures (300 K and 373 K) was calculated from these infrared reflectance spectra data using Equation (1) in Methods and summarized in Table 1. As the VO_2_ thickness decreases from 100 nm to 40 nm, the emissivity change of the multilayer structures increases from 0.31 to 0.52. Further decreasing the VO_2_ thickness below 30 nm decreases the Δε value. The largest change in normal emissivity of Δε ~ 0.52 was achieved from the multilayer structure with a 40 nm thick VO_2_ layer.

**Table 1:** Normal emittance calculated from the *experimentally measured* and *simulated* infrared reflectance spectra data using Equation (1) for multilayer structures (Si/VO_2_/BaF_2_/Au) with various VO_2_ thicknesses.

Thickness of VO_2_ layer (nm)	Experimentally measured			Simulated		
	ε_L_ (300 K)	ε_H_ (373 K)	Δε	ε_L_ (300 K)	ε_H_ (373 K)	Δε
40	0.15	0.64	0.49	0.12	0.64	0.52
60	0.16	0.63	0.47	0.14	0.59	0.45
80	0.21	0.61	0.40	0.17	0.54	0.37
100	0.25	0.55	0.30	0.18	0.49	0.31

The thickness of the BaF_2_ and Au layers was fixed at 1500 nm and 200 nm, respectively.

The effect of BaF_2_ thickness on the emissivity variation of the multilayer structures was also investigated using simulated reflectance spectra of the multilayer structures with BaF_2_ thickness of 600–1600 nm while the thickness of VO_2_ and Au was kept at 40 nm and 200 nm, respectively. As seen in Supplementary Fig. S1a, at 300 K as the BaF_2_ thickness increases from 600 to 1600 nm, the reflectance spectra of all samples are almost the same in the mid-infrared range (8–12 µm). However, at 373 K (Supplementary Fig. S1b), the reflectance spectra in the mid-infrared region gradually decreases with increasing BaF_2_ thickness. Thus, the overall Δε value increases slightly from 0.47 to 0.51 with the BaF_2_ thickness up to 1200 nm, where it saturates with a further increase to 1600 nm. (See Supplementary Table S1).

The hemispherical total emittance was numerically determined using Equation (2) in Methods. For a 60 nm VO_2_ film, the emittance at 300 K, ε_L_, was calculated to be 0.15 and the emittance at 373 K, ε_H_, was 0.54. It should be noted that the predicted change in total hemispherical emittance (Δε_hemi_) between 300 K and 373 K is 0.39, which is less than the normal emittance change (Δε = 0.45) (Table 1). The 60 nm sample was selected for hemispherical thermal power measurements as a compromise between performance and ease of fabrication.

### Experimental reflectance of multilayer structures

**Figure 5:**
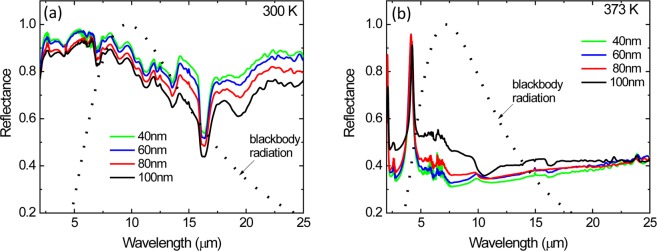
*Experimental* reflectance spectra of VO_2_-based multilayer structures with various VO_2_ thicknesses (40, 60, 80, and 100 nm) at (**a**) 300 K and (b) 373 K.

Figure 5 shows the variation of measured reflectance spectra in the infrared wavelength region (2–25 µm) for these multilayer structures (Si/VO_2_/BaF_2_/Au) at two different temperatures (300 and 373 K). At 300 K, since the VO_2_ is insulating and both the VO_2_ and BaF_2_ layers are transparent in the mid-infrared range (Fig. 5a), the multilayer structure behaves like a simple metal mirror resulting in a low emittance of the structure. However, at 373 K, the VO_2_ layer becomes metallic and semi-transparent in the infrared (Fig. 5b), which creates an interference filter with the BaF_2_ spacer and underlying Au layer, resulting in high emittance (i.e., low reflectance) of the multilayer structure. The BaF_2_ films were measured via atomic force microscopy to have an RMS roughness of 40 nm corresponding to an optical flatness of λ/250 at 10 μm. The optical path error in our multilayer radiators due to BaF_2_ roughness results in an intensity error of less than 1%. As seen in Table 1, as the VO_2_ film thickness decreases from 100 nm to 40 nm, the emittance change (Δε) of the multilayer structures increases from 0.30 to 0.49 due to a decrease in reflectance spectra of the multilayer structures. In this work, the largest normal emittance change of ~0.49 was observed from the multilayer structure with a 40 nm thick VO_2_ film. These experimental results agree well with the simulation results (see Table 1).

### Radiated thermal power measurements

As previously mentioned, hemispherical emittance is required to accurately calculate the radiated heat flux. Computation of hemispherical emittance from reflection data requires measurements at all angles of incidence, polarizations, and temperature. However, the indirect emittance measurements using reflectivity (*R*) data are experimentally prone to error and are difficult to achieve at near grazing angles. As an alternative, we performed calorimetric radiated power measurements as these are direct measurements of power radiated and do not require parameter retrieval from integration^30,31^.

**Figure 6:**
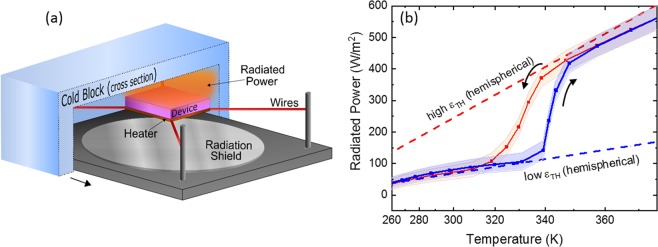
(**a**) A schematic illustration of radiated thermal power measurement setup in a vacuum chamber. (**b**) Radiated thermal power of a multilayer structure with a 60 nm thick VO_2_ layer. The direction of heating and cooling are indicated by arrows. The red and blue dashed lines represent the theoretical emitting power obtained from the simulated hemispherical emittance (ε) data with the insulating and metallic VO_2,_ respectively.

Figure 6(a) shows a schematic illustration of the radiated thermal power measurement setup which is housed inside a vacuum chamber. Figure 6(b) shows the experimentally measured emitted power of the multilayer radiators as a function of temperature. As the device temperature increases, the emitted power increases as described by the Stefan-Boltzmann law, P = σεAT ^4^, where σ is the Stefan-Boltzmann constant, ε is the total emittance, A is the surface area, and T is the absolute surface temperature. The cold block was held at a temperature of 108 K and the radiated power of the device was determined from the amount of electrical power required to maintain the device at a given temperature. For comparison, the theoretical emitting powers, calculated from the simulated total hemispherical emittances of the multilayer radiator with the insulating and metallic VO_2_ phases, are also plotted in Fig. 6(b) (See Methods section). The hemispherical emittance values were extracted by fitting the measured experimental data, resulting in ε_L_ ~ 0.16 and ε_H_ ~ 0.51. The sharp increase in radiated power around T~ 340 K is due to the change in emissivity across the phase transition of VO_2_.

The measured radiated power at 300 K and 373 K was 72 W/m^2^ and 552 W/m^2^, respectively. These results are consistent with a similar theoretical VO_2_-based design that took hemispherical emissivity into account^27^. Because the emitted power must be compared at two different temperatures, the measured emitted power is expected to differ by T^4^; therefore, it is helpful to measure the emitted power of the switchable radiator and to compare it with a radiator of fixed emissivity. This is accomplished by calculating the emitted power at 373 K using the experimentally determined hemispherical emissivity of 0.16. The predicted radiated power of a device without a phase transition is 175 W/m^2^ (for ε_L_~ 0.16 at 373 K), whereas the experimentally measured radiated power for the multilayer device is 552 W/m^2^. A summary of the normal and hemispherical emissivities and emitted power are shown in Table 2. Overall, the experimental results agree well with our simulated results, to within ±~10% error. This dramatic change in radiated power enables a spacecraft radiator to expel more heat as its temperature rises above *T*_*C*_, resulting in a net cooling effect. As the spacecraft temperature decreases below *T*_*C*_, the emissivity of the radiator decreases, thereby emitting less power, resulting in lower heat loss of the spacecraft. Thus, this multilayer structure provides an efficient, self-regulating, and passive method of switching the radiated power emissivity between the insulating and metallic states of VO_2_ layers.

**Table 2:** Summary of normal and hemispherical emittance and power for the multilayer radiator (Si/VO_2_/BaF_2_/Au) with a 60 nm VO_2_ layer at 300 K and 373 K.

Emissivity	Method	ε_L_ (300 K)	ε_H_ (373 K)	Δε	Power at 300 K (W/m^2^)	Power at 373 K (W/m^2^)	ΔP (W/m^2^)
Normal	Theory	0.14	0.59	0.45	63	677	614
	Experimental	0.16	0.63	0.47	72	691	619
Hemispherical	Theory	0.15	0.54	0.39	68	590	522
	Experimental	0.16	0.51	0.35	72	552	480

## Conclusion

We have experimentally demonstrated a passive radiator for thermal control in a simulated space environement via direct calorimetric measurments. The VO_2_-based thermal radiator, which consists of a BaF_2_ dielectric spacer sandwiched between a VO_2_ layer and an Au reflecting layer, achieves passive and switchable thermal emissivity control based on the VO_2_ phase transition. When the VO_2_ layer is in an insulating state at T <340 K, the multilayer structure behaves like a simple infrared reflector, thereby minimizing radiative heat loss. However, when the VO_2_ layer switches to a metallic state at T >340 K, the multilayer structure behaves like a resonant absorber with high emissivity around the mid-infrared region (8–12 µm), thus providing a radiative cooling effect. A quantitative comparison between the emitted power using normal and hemispherical emissivity is presented. The radiator shows an excellent switchability of Δε ~0.47 comparable with previously published results using the normal emissivity values. However, using normal emissivity values leads to an overestimate of actual performance because it does not take into account the angular dependence of the multilayer radiator design. While the measured hemispherical emissivity change of the radiator was Δε ~ 0.35, the experimentally measured radiated power increases by more than a factor 7 with a net radiated power difference of 480 W/m^2^ between 300 K and 373 K. This large and reversible emissivity change makes this design well suited for spacecraft thermal control applications.

## Methods

### Radiator structure

The device design presented here consists of a three layer stack of VO_2_, dielectric BaF_2_ and Au films. First, VO_2_ films (40–100 nm) were deposited on (100) Si substrates (>1000 Ωcm) at T ~773 K in 12 mTorr of oxygen via pulsed laser deposition using a KrF excimer laser (LPX 300, 248 nm, pulse duration of 20 ns) from a stoichiometric VO_2_ target (ACI Alloys)^29,32–34^. BaF_2_ dielectric layers (1500 nm) were deposited on top of the VO_2_ layer by electron-beam evaporation. Finally, 200 nm thick Au layers were deposited on the BaF_2_ layer by electron-beam evaporation. Figure 1(a) shows a schematic illustration of the multilayer structure mounted upside down on the temperature controlled stage inside a FTIR [JASCO]. IR illumination is directed into the Si substrate.

### Material characterization

X-ray diffraction (XRD) [Rigaku rotating anode X-ray generator with Cu K_α_ radiation] was used to characterize the crystal structure of the VO_2_ films. The electrical properties of VO_2_ films were measured by a four-point probe method at temperatures between 300 and 373 K. The optical reflectance spectra in the 2–25 µm wavelength range were measured at near normal incidence (10° AOI) at two temperatures (300 and 373 K) using an FTIR. The simulation studies were performed using the WVASE32 software (Woollam). The optical constants (refractive index *n*, extinction coefficient *k*) of the Au and Si substrates were obtained from the software database. The optical constants of VO_2_ and BaF_2_ layers were derived from ellipsometry measurements (IR-Vase, J. A. Woollam). A 200 nm thick Au film grown on a Si substrate was used as a reference. The roughness of the BaF_2_ films was measured by atomic force microscopy (Dimension Icon AFM, Bruker).

### Emittance and radiated power calculation

The normal emittance of the multilayer devices was calculated from the reflectance spectra data by using Kirchhoff’s law, with the spectral emittance ε_λ_, defined as ε(λ) =_d_1 – *R*(λ) for an opaque material, summed over the blackbody spectrum using the relation:

1 $${\rm{\varepsilon }}(\lambda )=\frac{{\int }_{{\lambda }_{1}}^{{\lambda }_{2}}(1-R(\lambda ,T))J(\lambda ,T)d\lambda }{{\int }_{{\lambda }_{1}}^{{\lambda }_{2}}J(\lambda ,T)d\lambda }$$

where *R(λ, T)* is the spectral reflectance at temperature *T* and *J(λ, T)* is the spectral blackbody radiation at temperature *T* given by Planck’s function for a given wavelength and temperature. The integral limits of λ_1_ and λ_2_ are 2 µm and 25 µm, respectively, as the blackbody radiation at room temperature is very weak beyond this range.

The hemispherical total emittance (ε_hemi_) was calculated from the following equation^35^:

2 $${\varepsilon }_{hemi}=\frac{1}{\sigma {T}^{4}}{\int }_{\varphi =0}^{2\pi }{\int }_{\theta =0}^{\pi /2}{\int }_{2\mu m}^{25\mu m}[\varepsilon (\lambda ,\theta ,\varphi ,T)J(\lambda ,T)d\lambda ]\,\cos \,\theta \,\sin \,\theta \,d\theta \,d\varphi \,$$

where *ε(λ*, θ, ɸ, *T)* is the directional spectral emissivity at temperature *T* and at an emission angle of *θ* and *ɸ*.

The total hemispherical emitted power was calculated from the following equation:

3 $${{\rm{P}}}_{{\rm{rad}}}=\sigma {{\rm{\varepsilon }}}_{{\rm{hemi}}}A\,{T}^{{\rm{4}}}$$

where σ is the Stefan-Boltzmann constant, ε_hemi_ is the hemispherical total emittance, *A* is the surface area, and *T* is the absolute surface temperature.

### Direct radiated power measurements

The performance of the radiators was measured in a vacuum chamber by mounting a heater to the gold side of the radiator and placing a cold block ~1 cm from the surface of the radiator simulating the coldness of space (blackbody temperature ~ 108 K) as shown in Fig. 6a. The power delivered to the heater was varied while monitoring the sample temperature. Convective losses are assumed to be zero because the experiments were performed in vacuum (1 × 10^−7^ Torr). The conductive losses are small due to the high thermal isolation between the sample/heater and have been calibrated out of the test fixture. Therefore, the emitted power and change in emissivity can be determined directly from the power delivered to the heater.

## Supplementary information

Supplementary Information
