# Supplementary material for: VO2-based switchable radiator for spacecraft thermal control
Source: Sci Rep. 2019 Aug 5;9:11329. doi: 10.1038/s41598-019-47572-z (PMC6683125; doi:10.1038/s41598-019-47572-z)
Supplement: Supplementary file 1 — Supplementary Information [file 41598_2019_47572_MOESM1_ESM.pdf]

Supplementary information for

"VO_2-based switchable radiator for spacecraft thermal control"

Heungsoo Kim, Kwok Cheung, Raymond C.Y. Auyeung, Donald E. Wilson, Kristin M. Charipar, Alberto Piqué, and Nicholas A. Charipar

Naval Research Laboratory, Washington, DC 20375, USA

Simulated reflectance spectra as a function of BaF_2 spacer thickness

In the main manuscript we have shown the simulated reflectance spectra of the multilayer structure ($Si/VO_2/BaF_2/Au$) as a function of VO_2 film thickness. Here, we provide the simulated reflectance spectra of the multilayer structure ($Si/VO_2/BaF_2/Au$) as a function of BaF_2 layer thickness (600 - 1600 nm) while the thickness of VO_2 and Au was kept at 40 nm and 200 nm, respectively. As seen in Figure S1, at 300 K, as the BaF_2 thickness increases from 600 to 1600 nm, the overall reflectance of multilayer structures is almost the same in the mid-infrared range (8-12 μm) for all samples. However, at 373 K the reflectance spectrum in the mid-infrared region decreases with increasing BaF_2 thickness, resulting in increased emittance. The emittance variation ($\Delta\varepsilon$) of these multilayer structures between these two temperatures (300 K and 373 K) was calculated from these simulated reflectance spectra data using equation (1) in Methods and summarized in Table S1. As the BaF_2 thickness increases from 600 nm to 1200 nm, the overall $\Delta\varepsilon$ in the infrared spectrum (2 - 25 μm) increases slightly from 0.47 to 0.51 and then saturates with increasing BaF_2 thickness up to 1600 nm. Thus, 1200 – 1600 nm is the optimum BaF_2 thickness range to achieve the maximum interference effect in the mid-infrared region (8 - 12 μm), providing a high emittance change between 300 K and 373 K.

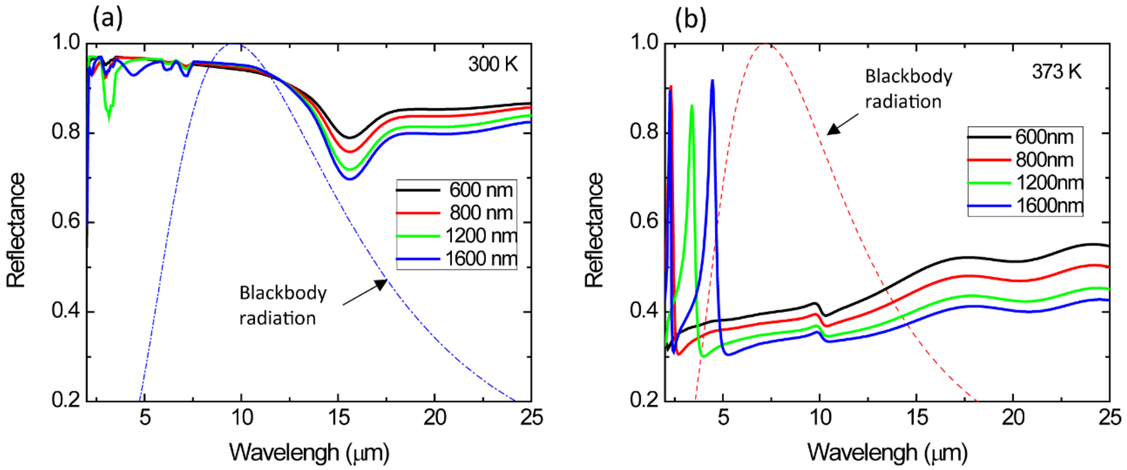

Figure S1. Simulated reflectance spectra of VO_2-based multilayer structures with various BaF_2 thicknesses (600, 800, 1200, and 1600 nm) at (a) 300 K and (b) 373 K while the thickness of the VO_2 and Au was kept at 40 nm and 200 nm, respectively.

Table S1. Emittance calculated from the *simulated* infrared reflectance spectra data for multilayer structures ($Si/VO_2/BaF_2/Au$) with various BaF_2 thicknesses. The thickness of the VO_2 and Au layers was fixed at 40 nm and 200 nm, respectively.

Thickness of BaF_2 layer (nm)	ε_L(300 K)	ε_H(373 K)	$\Delta\varepsilon$
600	0.10	0.57	0.47
800	0.11	0.60	0.49
1200	0.12	0.63	0.51
1600	0.13	0.64	0.51
